# Predictive factors of permanent hypothyroidism after subacute thyroiditis: a systematic review and meta-analysis

**DOI:** 10.3389/fendo.2026.1720417

**Published:** 2026-04-21

**Authors:** Qianhui Cui, Meng Wang, Jingqiu Cui

**Affiliations:** 1 Tianjin Medical University, Tianjin, China; 2 Department of Endocrinology and Metabolism, Tianjin Medical University General Hospital, Tianjin, China

**Keywords:** meta-analysis, permanent hypothyroidism, predictive factors, subacute thyroiditis, systematic review

## Abstract

**Background:**

Subacute thyroiditis (SAT) is a self-limiting thyroid inflammatory disorder, but some patients develop permanent hypothyroidism, impacting long-term health. We aimed to systematically synthesize evidence on factors associated with permanent hypothyroidism after SAT.

**Methods:**

A systematic search of PubMed, Embase, the Cochrane Library and Web of Science was conducted up to August 2025. Randomized trials, cohort, and case-control studies evaluating predictors of permanent hypothyroidism after SAT were included. Data were extracted following PRISMA guidelines, and study quality was assessed using the Newcastle–Ottawa Scale (NOS). Random-effects models were preferentially applied given expected clinical heterogeneity, with heterogeneity quantified using I^2^ and τ^2^.

**Results:**

Ten studies involving 1294 patients were included. Higher free triiodothyronine (FT3) levels (mean difference = 1.85, 95% CI: 0.60–3.09; Z = 2.91, P = 0.004; I² = 0%) and positive thyroglobulin antibodies (TgAb) (OR = 2.57, 95% CI: 1.35–4.88, P = 0.004; I² = 57%, τ² = 0.13) were significantly associated with an increased risk of permanent hypothyroidism after SAT. Corticosteroid therapy was associated with a lower odds of permanent hypothyroidism compared with NSAID-based management (OR = 0.40, 95% CI: 0.23–0.70, P = 0.001; I² = 36%).

**Conclusions:**

FT3, TgAb positivity and treatment modality are associated with the risk of permanent hypothyroidism following SAT. Compared with NSAID-based management, corticosteroid therapy is associated with lower odds of developing permanent hypothyroidism. These findings support early identification of high-risk patients and personalized follow-up strategies.

**Systematic review registration:**

https://www.crd.york.ac.uk/prospero/, identifier CRD420251064643.

## 1 Introduction

Subacute thyroiditis (SAT) is a common type of thyroid inflammatory disease, accounting for approximately 5%**–**10% of all thyroid disorders (1). Its onset is usually associated with viral infections (2). Proposed mechanisms include virus-triggered follicular destruction, activation of innate and adaptive immune responses, and underlying autoimmune susceptibility (3). The typical clinical manifestations include tenderness in the thyroid area, radiating pain, fever, and characteristic changes in thyroid function: first is the thyrotoxic phase, followed by the hypothyroid phase, and eventually the recovery phase (4). Although most patients can recover spontaneously within several months to a year, approximately 5%**–**30% may develop permanent hypothyroidism (5), requiring lifelong thyroid hormone replacement therapy. Therefore, early identification and intervention for high-risk patients are crucial for improving long-term prognosis.

In recent years, numerous studies have explored the predictive factors for the development of permanent hypothyroidism following SAT, covering demographic characteristics (6), clinical indicators (7), laboratory parameters (8) and treatment methods (9), etc. However, most are single-center, retrospective, and underpowered, with heterogeneous outcome definitions and treatment strategies.

This study aims to integrate current evidence through systematic review and meta-analysis to identify independent predictive factors of permanent hypothyroidism after SAT and to quantify their effect sizes, thereby providing evidence for clinical risk stratification and management. The study protocol was prospectively registered in PROSPERO (CRD420251064643), and no major deviations from the registered protocol occurred.

## 2 Methods

### 2.1 Search strategy

A comprehensive search was conducted in PubMed, Embase, the Cochrane Library and Web of Science to identify relevant studies published up to August 2025. Search terms included combinations of subject headings and free-text terms such as “subacute thyroiditis” “hypothyroidism” “randomized controlled trials” “case-control studies” and “cohort studies”. Full electronic search strategies for each database are provided in **Supplementary Table 1**. No language restrictions were applied, and preprints were excluded.

### 2.2 Selection criteria

Inclusion criteria: (1) Studies enrolling patients with a confirmed diagnosis of SAT, based on typical clinical manifestations, laboratory findings, and/or imaging features (10); (2) Studies reporting follow-up thyroid function outcomes after the acute phase of SAT and evaluating potential factors associated with the development of persistent or permanent hypothyroidism; (3) Studies providing a clear definition of persistent or permanent hypothyroidism, with a minimum follow-up duration of at least six months after disease onset (11).

Exclusion criteria: (1) Duplicate publications or studies with overlapping populations; (2) Non-original studies, including reviews, case reports, editorials, or conference abstracts; (3) Studies with insufficient or non-extractable data for quantitative synthesis; (4) Studies with a follow-up duration shorter than six months or without a clearly defined thyroid function outcome.

The definition of permanent hypothyroidism varied across the included studies. For the purpose of this meta-analysis, we accepted the original outcome definitions used in each study, while applying a minimum follow-up duration of six months after the acute phase of SAT. This time threshold reflects the most commonly adopted follow-up duration in the existing literature, rather than a novel definition proposed by the authors.

### 2.3 Data extraction and quality assessment

Two reviewers independently extracted data using a standardized form, and discrepancies were resolved through discussion with a third reviewer. Extracted data included: (1) Basic study information (e.g., first author, publication year, study location, study design and follow-up duration); (2) Participant characteristics (e.g., sample size, age and sex distribution); (3) Incidence of permanent hypothyroidism; (4) Specific data and effect estimates of each predictive factor (e.g., odds ratio, hazard ratio and 95% confidence intervals). The quality of the included studies was assessed using the Newcastle–Ottawa Scale (NOS) (12). This scale evaluates studies based on three dimensions: selection of study participants, comparability between groups, and outcome ascertainment. The total score ranges from 0 to 9, with studies scoring ≥7 considered to be of high quality and at low risk of bias (13).

### 2.4 Statistical analysis

Statistical analysis was performed using RevMan 5.4 and STATA 16.0. Odds ratios (ORs) with 95% confidence intervals (CIs) were pooled. Random-effects models were used preferentially; fixed-effects models were applied only when heterogeneity was negligible. For each analysis, I^2^, Cochran’s Q-test P values, and τ^2^ (when applicable) are reported. Sensitivity analyses were conducted by sequentially excluding individual studies. Potential publication bias was examined using Egger’s test in combination with funnel plots.

## 3 Results

### 3.1 Search results and study characteristics

**Figure 1:**
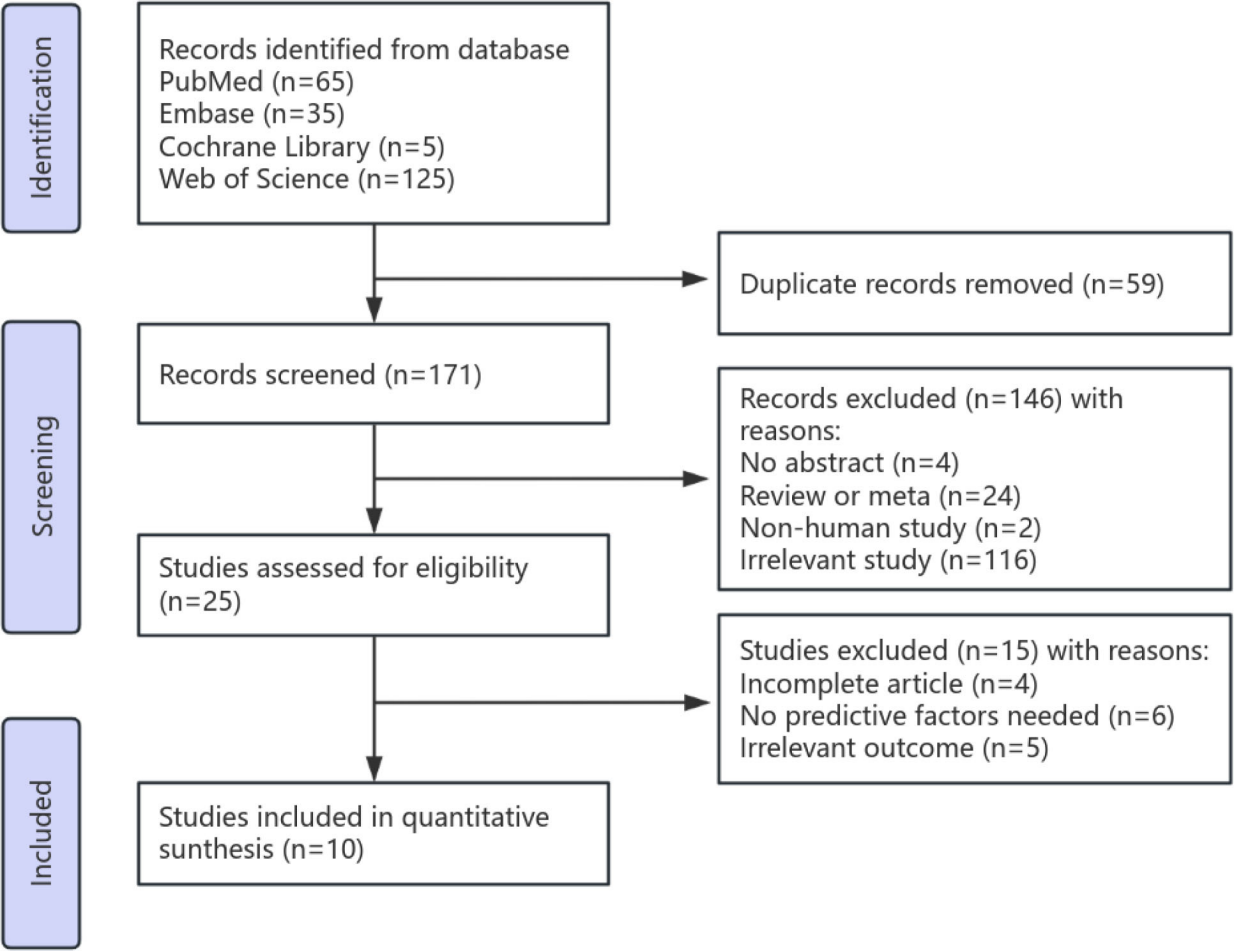
PRISMA 2020 flow diagram.

The literature selection process is presented in **Figure 1**. A total of 230 relevant articles were initially retrieved. After the removal of duplicates (n = 59), 171 records underwent title and abstract screening, during which 146 were excluded for not meeting the inclusion criteria. The remaining 25 articles were assessed in full text, and ultimately, 10 studies met the eligibility criteria and were included in this meta-analysis (5, 11, 14–21). All 10 studies were cohort designs, encompassing a total of 1,294 patients. The included studies involved populations from diverse regions, including Asia and Europe. In each study, the diagnoses of SAT and permanent hypothyroidism were clearly defined. The baseline characteristics of the included studies are summarized in **Table 1**.

**Table 1:** Basic information of included studies.

Name	Year	Country	N (Male/Female)	Age (years)	Treatment	Definition of permanent hypothyroidism				Follow-up (months)	With/without hypothyroidism
						TSH threshold	Duration	Treatment status or not	Observation time point		
Omori	2008	Japan	32(3/29)	44.20 ± 9.40	NSAIDs/PSL	–	–	Need to continue LT4 replacement	Continuous treatment status	–	2/27
Nishihara	2009	Japan	252(26/226)	48.60 ± 9.50	None/NSAIDs/PSL	Rise	At least 3 months	Need to continue LT4 replacement	Calculated from the first TSH rise	21.1 ± 17.3	135/117
Schenke	2013	Germany	72(13/59)	49.00 ± 11.00	NSAIDs/corticosteroids/combination	>3.5 mU/L	–	–	Follow-up period	≥12	19/53
Sencar	2019	Turkey	217(40/177)	43.00 ± 9.00	NSAIDs/Methylprednisolone	–	At least 6 months	Need to continue LT4 replacement	SAT hypothyroidism lasting ≥6 months	27(6.2-64)	27/190
Görges	2019	Germany	127(19/108)	47.60 ± 11.00	NSAIDs/PSL/combination	–	At least 1 year	Need to continue LT4 replacement	1 year after initial visit	–	34/93
Sencar	2020	Turkey	247(63/184)	44.00 ± 7.50	Low-dose methylprednisolone	–	At least 6 months	Need to continue LT4 replacement	SAT hypothyroidism lasting ≥6 months	29(6.2-70)	24/220
Zhao	2020	China	61(15/46)	44.07 ± 9.20	NSAIDs/PSL/combination	TSH > 10 mIU/L (asymptomatic) or symptomatic + TSH < 10 mIU/L	At least 2 years	Need to continue LT4 replacement therapy	End of 2-year follow-up period	24	20/41
Gokkaya	2024	Turkey	51(19/32)	42.88 ± 9.14	NSAIDs/Corticosteroids	TSH > 10 mIU/L (asymptomatic)	At least 1 year	Need to continue LT4 replacement therapy	More than 1 year after SAT diagnosis	48 (42–66)	16/35
Hacisahinogullari	2024	Turkey	96(28/68)	41.14 ± 8.84	NSAIDs/GCs/combination	–	At least 1 year	Overt or subclinical hypothyroidism, with or without treatment	1 year after initial SAT visit	12	27/69
Corsello	2025	Italy	139(28/111)	48.50	NSAIDs/GCs/combination	TSH > 4 μUI/mL	Lasts ≥6.5 months	TSH remains > 4 μUI/mL after LT4 dose reduction or discontinuation	Last follow-up	–	48/91

GCs, glucocorticoids; LT4, levothyroxine; NSAIDs, Non-Steroidal Anti-Inflammatory Drugs; PSL, prednisolone; SAT, subacute thyroiditis; TSH, thyroid-stimulating hormone.

### 3.2 Quality assessment

All studies included in this meta-analysis were assessed for quality and risk of bias using the NOS. The scores for individual studies are presented in **Table 2**, with an average score of 7.4 (range: 7-9). All studies were assessed as being of high quality and at low risk of bias.

**Table 2:** Quality evaluation of the eligible studies with the Newcastle-Ottawa Scale (range 0-9).

Study	Representativeness of the exposed cohort	Selection of the nonexposed cohort	Ascertainment of exposure	Outcome not present at the start	Comparability on the most important factor	Assessment of outcome	Long enough follow-up	Adequacy of follow-up of cohorts	Quality score
Omori	*	–	*	*	*	*	*	*	7
Nishihara	*	–	*	*	*	*	*	*	7
Schenke	*	–	*	*	*	*	*	*	7
Sencar	*	–	*	*	*	*	*	*	7
Görges	*	–	*	*	*	*	*	*	7
Sencar	*	–	*	*	*	*	*	*	7
Zhao	*	*	*	*	**	*	*	*	9
Gokkaya	–	*	*	*	*	*	*	*	7
Hacisahinogullari	–	*	*	*	*	*	*	*	7
Corsello	*	*	*	*	**	*	*	*	9

Each asterisk (*) represents 1 score.

### 3.3 Predictive factors for permanent hypothyroidism

For each pooled analysis, statistical heterogeneity was assessed using the I² statistic, and the choice of effect model was determined based on both statistical heterogeneity and anticipated clinical heterogeneity. Random-effects models were applied when I² exceeded 50% or when substantial clinical heterogeneity was expected due to differences in study populations, treatment strategies, or outcome definitions. When fixed-effects models were used despite low I² values, this decision was justified by consistent study designs and homogeneous effect estimates. For random-effects models, between-study variance (τ²) was additionally reported.

**Figure 2:**
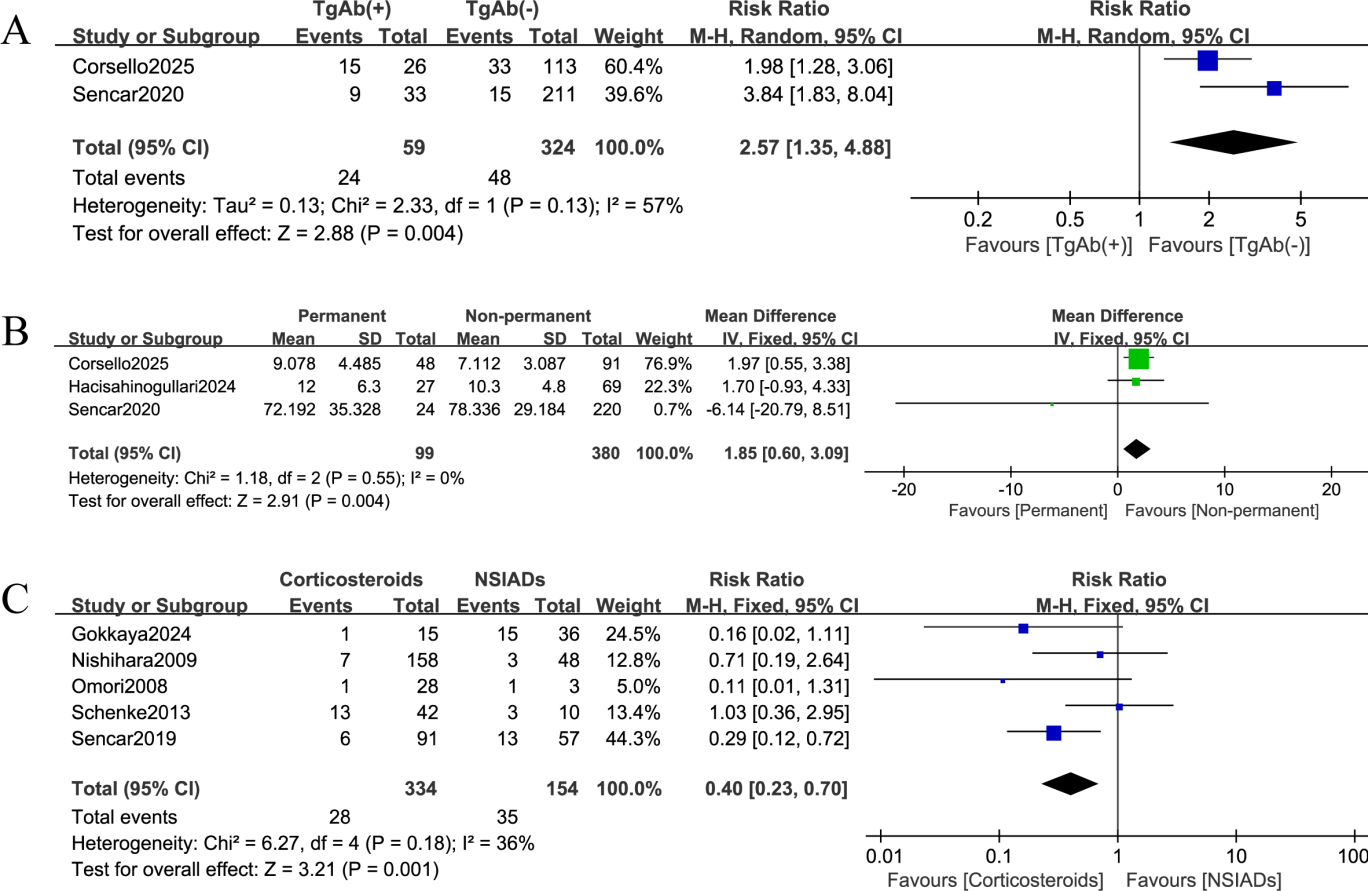
Forest plots of predictive factors for permanent hypothyroidism following subacute thyroiditis (SAT). **(A)** Association between thyroglobulin antibody (TgAb) positivity and risk of permanent hypothyroidism. **(B)** Association between baseline free triiodothyronine (FT3) level and risk of permanent hypothyroidism. **(C)** Association between treatment modality (non-steroidal anti-inflammatory drugs [NSAIDs] vs. corticosteroids) and risk of permanent hypothyroidism.

A total of two studies were included in the analysis of thyroglobulin antibodies (TgAb) status. Patients with a positive TgAb test had a significantly higher risk of developing permanent hypothyroidism (OR = 2.57, 95% CI: 1.35–4.88, P = 0.004; I² = 57%, τ² = 0.13; random-effects model; **Figure 2A**).

For the analysis of baseline FT3 levels (measured in pmol/L) as a continuous variable, three studies were included, for which the units of measurement had been manually harmonized to ensure consistency. The mean difference analysis showed that patients who developed permanent hypothyroidism had higher baseline FT3 levels than those who recovered (mean difference = 1.85, 95% CI: 0.60–3.09; Z = 2.91, P = 0.004; I² = 0%; **Figure 2B**). Five studies contributed to the comparison of treatment modalities. Corticosteroid therapy was associated with lower odds of permanent hypothyroidism compared with NSAID-based management (OR = 0.40, 95% CI: 0.23–0.70, P = 0.001; I² = 36%; **Figure 2C**). For both analyses, a fixed-effects model was applied as statistical heterogeneity was low and effect estimates were directionally consistent, despite anticipated clinical heterogeneity across studies.

### 3.4 Sensitivity analysis and publication bias

**Figure 3:**
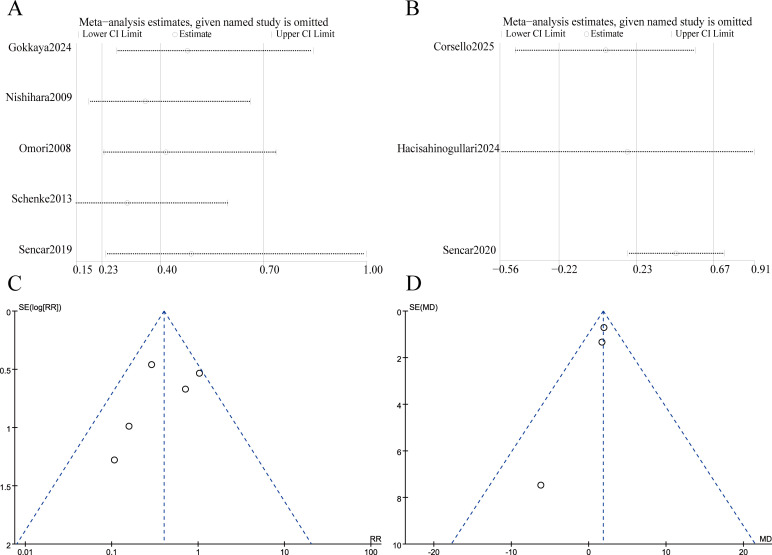
Sensitivity analysis and publication bias assessment. **(A)** Leave-one-out sensitivity analysis for non-steroidal anti-inflammatory drug (NSAID) treatment. **(B)** Leave-one-out sensitivity analysis for baseline free triiodothyronine (FT3) level. **(C)** Funnel plot for publication bias in studies of NSAID-based management versus corticosteroid therapy. **(D)** Funnel plot for publication bias in studies of baseline FT3 level.

To assess the reliability and consistency of this meta-analysis, sensitivity analyses and publication bias assessments were conducted for outcomes derived from three or more studies. Sensitivity analysis was conducted using the leave-one-out method. The findings showed that the link between NSAID treatment and the risk of permanent hypothyroidism is robust and not influenced by any single study. In contrast, the link between higher baseline FT3 levels and permanent hypothyroidism was less consistent, as the results varied with the exclusion of certain studies (**Figure 3**). Publication bias was assessed using funnel plot and Egger’s test. It should be noted that the statistical power of these tests is limited when the number of included studies is fewer than ten. No significant bias was found, with p-values of 0.435 and 0.589, respectively.

## 4 Discussion

Our meta-analysis identified positive TgAb status at diagnosis as a significant predictor of permanent hypothyroidism. This finding aligns with the established understanding of SAT as an immune-mediated process. TgAb positivity likely reflects a more robust or dysregulated autoimmune response directed against the thyroid gland, which may not only resolve the acute inflammation but also progress to persistent autoimmune thyroiditis and long-term functional impairment (22). Previous studies have linked the presence of TgAb to an increased risk of chronic thyroiditis and permanent thyroid damage (23, 24). Beyond confirming this association, our pooled analysis quantifies its predictive strength, reinforcing the clinical utility of TgAb testing. Assessing TgAb at initial diagnosis therefore provides valuable prognostic information for patient counseling and structuring long-term follow-up, potentially identifying a subgroup of patients who may benefit from closer monitoring. Monitoring changes in TgAb titers over time may further refine risk stratification (25).

Elevated baseline FT3 levels were also associated with an increased risk of permanent hypothyroidism, although this association was less stable in sensitivity analysis. This trend may be driven by more severe follicular destruction and robust inflammatory activity during the acute phase (26), which leads to irreversible loss of functional thyroid tissue and thereby increases the risk of permanent hypothyroidism. Our findings are consistent with previous reports suggesting that FT3 levels may serve as an early indicator of disease severity (27). However, further verification is necessary before applying FT3 measurement in clinical practice.

Regarding treatment, our analysis showed that patients receiving NSAIDs had a higher likelihood of developing permanent hypothyroidism compared with those treated with corticosteroids. This association may reflect the stronger anti-inflammatory effects of corticosteroids, which could potentially limit thyroid tissue damage during the inflammatory phase of SAT (28). However, the available evidence does not allow us to determine whether this effect interacts with immunological markers such as TgAb, and residual confounding cannot be excluded. In addition, TgAb positivity should be interpreted as a general risk marker for permanent hypothyroidism after SAT rather than a predictor restricted to a specific treatment modality.

Key strengths of this study include a systematic literature search with predefined inclusion criteria, quality assessment of included studies using the NOS, and sensitivity analyses to verify the robustness of the results. Furthermore, Egger’s test was employed to assess the potential publication bias. These methodological considerations strengthen the reliability of the findings.

Several limitations must also be acknowledged. The number of eligible studies was relatively small, with each having only a moderate sample size. The association between baseline FT3 levels and permanent hypothyroidism should be interpreted cautiously, as it was supported by a limited number of studies and lacked robustness in sensitivity analyses. Furthermore, potential confounding factors—including baseline thyroid reserve, genetic susceptibility, environmental influences, and variations in treatment protocols—could not be completely accounted for, which may influence the observed associations (29). Finally, owing to the wide heterogeneity in follow-up duration and the limited number of studies within potential strata, sensitivity analyses stratified by follow-up length were not performed. Additional clinically relevant predictors were considered; however, meta-analysis was not feasible due to the limited number of eligible studies.

Overall, the certainty of evidence for most predictors is moderate to low, reflecting the observational design of included studies, limited sample sizes, and residual heterogeneity.

In conclusion, TgAb positivity and treatment modality are consistently associated with the risk of permanent hypothyroidism after SAT, with NSAID-based management showing higher odds compared with corticosteroid therapy, while elevated baseline FT3 may be associated with increased risk but requires cautious interpretation. These findings provide clinically relevant insights for risk stratification and management. Importantly, incorporating these predictors into clinical practice could improve long-term outcomes by guiding personalized follow-up and treatment strategies.

## Data Availability

The original contributions presented in the study are included in the article/**Supplementary Material**. Further inquiries can be directed to the corresponding author.
