# Supplementary material for: Predictive factors of permanent hypothyroidism after subacute thyroiditis: a systematic review and meta-analysis
Source: Front Endocrinol (Lausanne). 2026 Apr 21;17:1720417. doi: 10.3389/fendo.2026.1720417 (PMC13138911; doi:10.3389/fendo.2026.1720417)
Supplement: Supplementary file 1 [file DataSheet1.docx]

Supplementary Material

# Supplementary Table 1

# Full electronic search strategies for each database.

| **MeSH** | **Entry Terms** |
| --- | --- |
| Thyroiditis, Subacute | Subacute Thyroiditi*  De Quervain Thyroiditis  Giant Cell Thyroiditi*  Granulomatous Thyroiditi*  Subacute Nonsuppurative Thyroiditi*  Subacute Painful Thyroiditi*  SAT |
| Hypothyroidism | Hypothyroidism*  Thyroid Stimulating Hormone Deficienc*  TSH Deficienc*  Secondary Hypothyroidism*  Central Hypothyroidism*  Primary Hypothyroidism* |
| Randomized Controlled Trials | Randomized Controlled Trial*  Randomized Clinical Trial*  Randomized Controlled Clinical Trial*  RCT |
| Case-Control Studies | Case Control Stud*  Case Comparison Stud*  Case Compeer Stud*  Case Base Stud*  Case Referent Stud*  Case Referrent Stud*  Matched Case Control Stud*  Nested Case Control Stud* |
| Cohort Studies | Cohort Stud*  Concurrent Stud*  Closed Cohort Stud*  Historical Cohort Stud*  Incidence Stud*  Cohort Analys*  Birth Cohort Stud* |

**PubMed-65**

| **Search number** | **Query** |
| --- | --- |
| #1 | "Thyroiditis, Subacute"[Mesh] |
| #2 | ((((((Subacute Thyroiditi*[Title/Abstract]) OR (De Quervain Thyroiditis[Title/Abstract])) OR (Giant Cell Thyroiditi*[Title/Abstract])) OR (Granulomatous Thyroiditi*[Title/Abstract])) OR (Subacute Nonsuppurative Thyroiditi*[Title/Abstract])) OR (Subacute Painful Thyroiditi*[Title/Abstract])) OR (SAT[Title/Abstract]) |
| #3 | #1 OR #2 |
| #4 | "Hypothyroidism"[Mesh] |
| #5 | (((((Hypothyroidism*[Title/Abstract]) OR (Thyroid Stimulating Hormone Deficienc*[Title/Abstract])) OR (TSH Deficienc*[Title/Abstract])) OR (Secondary Hypothyroidism*[Title/Abstract])) OR (Central Hypothyroidism*[Title/Abstract])) OR (Primary Hypothyroidism*[Title/Abstract]) |
| #6 | #4 OR #5 |
| #7 | (("Randomized Controlled Trial" [Publication Type]) OR "Case-Control Studies"[Mesh]) OR "Cohort Studies"[Mesh] |
| #8 | ((((((((((((((((((Randomized Controlled Trial*[Title/Abstract]) OR (Randomized Clinical Trial*[Title/Abstract])) OR (Randomized Controlled Clinical Trial*[Title/Abstract])) OR (RCT[Title/Abstract])) OR (Case Control Stud*[Title/Abstract])) OR (Case Comparison Stud*[Title/Abstract])) OR (Case Compeer Stud*[Title/Abstract])) OR (Case Base Stud*[Title/Abstract])) OR (Case Referent Stud*[Title/Abstract])) OR (Case Referrent Stud*[Title/Abstract])) OR (Matched Case Control Stud*[Title/Abstract])) OR (Nested Case Control Stud*[Title/Abstract])) OR (Cohort Stud*[Title/Abstract])) OR (Concurrent Stud*[Title/Abstract])) OR (Closed Cohort Stud*[Title/Abstract])) OR (Historical Cohort Stud*[Title/Abstract])) OR (Incidence Stud*[Title/Abstract])) OR (Cohort Analys*[Title/Abstract])) OR (Birth Cohort Stud*[Title/Abstract]) |
| #9 | #7 OR #8 |
| #10 | #3 AND #6 AND #9 |

**Embase-35**

| **Search number** | **Query** |
| --- | --- |
| #1 | 'subacute thyroiditis'/exp |
| #2 | 'subacute thyroiditi*':ti,ab,kw OR 'de quervain thyroiditis':ti,ab,kw OR 'giant cell thyroiditi*':ti,ab,kw OR 'granulomatous thyroiditi*':ti,ab,kw OR 'subacute nonsuppurative thyroiditi*':ti,ab,kw OR 'subacute painful thyroiditi*':ti,ab,kw OR sat:ti,ab,kw |
| #3 | #1 OR #2 |
| #4 | 'hypothyroidism'/exp |
| #5 | hypothyroidism*:ti,ab,kw OR 'thyroid stimulating hormone deficienc*':ti,ab,kw OR 'tsh deficienc*':ti,ab,kw OR 'secondary hypothyroidism*':ti,ab,kw OR 'central hypothyroidism*':ti,ab,kw OR 'primary hypothyroidism*':ti,ab,kw |
| #6 | #4 OR #5 |
| #7 | 'randomized controlled trial (topic)'/exp OR 'case control study'/exp OR 'cohort analysis'/exp |
| #8 | 'randomized controlled trial*':ti,ab,kw OR 'randomized clinical trial*':ti,ab,kw OR 'randomized controlled clinical trial*':ti,ab,kw OR rct:ti,ab,kw OR 'case control stud*':ti,ab,kw OR 'case comparison stud*':ti,ab,kw OR 'case compeer stud*':ti,ab,kw OR 'case base stud*':ti,ab,kw OR 'case referent stud*':ti,ab,kw OR 'case referrent stud*':ti,ab,kw OR 'matched case control stud*':ti,ab,kw OR 'nested case control stud*':ti,ab,kw OR 'cohort stud*':ti,ab,kw OR 'concurrent stud*':ti,ab,kw OR 'closed cohort stud*':ti,ab,kw OR 'historical cohort stud*':ti,ab,kw OR 'incidence stud*':ti,ab,kw OR 'cohort analys*':ti,ab,kw OR 'birth cohort stud*':ti,ab,kw |
| #9 | #7 OR #8 |
| #10 | #3 AND #6 AND #9 |

**Cochrane-5**

| **Search number** | **Query** |
| --- | --- |
| #1 | MeSH descriptor: [Thyroiditis, Subacute] explode all trees |
| #2 | (Subacute Thyroiditi*):ti,ab,kw OR (De Quervain Thyroiditis):ti,ab,kw OR (Giant Cell Thyroiditi*):ti,ab,kw OR (Granulomatous Thyroiditi*):ti,ab,kw OR (Subacute Nonsuppurative Thyroiditi*):ti,ab,kw OR (Subacute Painful Thyroiditi*):ti,ab,kw OR (SAT):ti,ab,kw |
| #3 | #1 OR #2 |
| #4 | MeSH descriptor: [Hypothyroidism] explode all trees |
| #5 | (Hypothyroidism*):ti,ab,kw OR (Thyroid Stimulating Hormone Deficienc*):ti,ab,kw OR (TSH Deficienc*):ti,ab,kw OR (Secondary Hypothyroidism*):ti,ab,kw OR (Central Hypothyroidism*):ti,ab,kw OR (Primary Hypothyroidism*):ti,ab,kw |
| #6 | #4 OR #5 |
| #7 | MeSH descriptor: [Randomized Controlled Trial] explode all trees |
| #8 | MeSH descriptor: [Case-Control Studies] explode all trees |
| #9 | MeSH descriptor: [Cohort Studies] explode all trees |
| #10 | #7 OR #8 OR #9 |
| #11 | (Randomized Controlled Trial*):ti,ab,kw OR (Randomized Clinical Trial*):ti,ab,kw OR (Randomized Controlled Clinical Trial*):ti,ab,kw OR (RCT):ti,ab,kw OR (Case Control Stud*):ti,ab,kw OR (Case Comparison Stud*):ti,ab,kw OR (Case Compeer Stud*):ti,ab,kw OR (Case Base Stud*):ti,ab,kw OR (Case Referent Stud*):ti,ab,kw OR (Case Referrent Stud*):ti,ab,kw OR (Matched Case Control Stud*):ti,ab,kw OR (Nested Case Control Stud*):ti,ab,kw OR (Cohort Stud*):ti,ab,kw OR (Concurrent Stud*):ti,ab,kw OR (Closed Cohort Stud*):ti,ab,kw OR (Historical Cohort Stud*):ti,ab,kw OR (Incidence Stud*):ti,ab,kw OR (Cohort Analys*):ti,ab,kw OR (Birth Cohort Stud*):ti,ab,kw |
| #12 | #10 OR #11 |
| #13 | #3 AND #6 AND #12 |

**WOS-125**

| **Search number** | **Query** |
| --- | --- |
| #1 | Subacute Thyroiditi* (Topic) or De Quervain Thyroiditis (Topic) or Giant Cell Thyroiditi* (Topic) or Granulomatous Thyroiditi* (Topic) or Subacute Nonsuppurative Thyroiditi* (Topic) or Subacute Painful Thyroiditi* (Topic) or SAT (Topic) and Preprint Citation Index (Exclude – Database) |
| #2 | Hypothyroidism* (Topic) or Thyroid Stimulating Hormone Deficienc* (Topic) or TSH Deficienc* (Topic) or Secondary Hypothyroidism* (Topic) or Central Hypothyroidism* (Topic) or Primary Hypothyroidism* (Topic) and Preprint Citation Index (Exclude – Database) |
| #3 | Randomized Controlled Trial* (Topic) or Randomized Clinical Trial* (Topic) or Randomized Controlled Clinical Trial* (Topic) or RCT (Topic) or Case Control Stud* (Topic) or Case Comparison Stud* (Topic) or Case Compeer Stud* (Topic) or Case Base Stud* (Topic) or Case Referent Stud* (Topic) or Case Referrent Stud* (Topic) or Matched Case Control Stud* (Topic) or Nested Case Control Stud* (Topic) or Cohort Stud* (Topic) or Concurrent Stud* (Topic) or Closed Cohort Stud* (Topic) or Historical Cohort Stud* (Topic) or Incidence Stud* (Topic) or Cohort Analys* (Topic) or Birth Cohort Stud* (Topic) and Preprint Citation Index (Exclude – Database) |
| #4 | #1 AND #2 AND #3 |

# Supplementary Table 2

**PRISMA 2020 checklist.**

| **Section and Topic** | **Item #** | **Checklist item** | **Location where item is reported** |
| --- | --- | --- | --- |
| **TITLE** | | |  |
| Title | 1 | Identify the report as a systematic review. | A Systematic Review and Meta-Analysis |
| **ABSTRACT** | | |  |
| Abstract | 2 | See the PRISMA 2020 for Abstracts checklist. | Abstract section (Background, Methods, Results, Conclusions) |
| **INTRODUCTION** | | |  |
| Rationale | 3 | Describe the rationale for the review in the context of existing knowledge. | Introduction, first and second paragraphs |
| Objectives | 4 | Provide an explicit statement of the objective(s) or question(s) the review addresses. | Introduction, “This study aims to integrate current evidence... to identify independent predictive factors...” |
| **METHODS** | | |  |
| Eligibility criteria | 5 | Specify the inclusion and exclusion criteria for the review and how studies were grouped for the syntheses. | Methods, 3.2 Selection criteria |
| Information sources | 6 | Specify all databases, registers, websites, organisations, reference lists and other sources searched or consulted to identify studies. Specify the date when each source was last searched or consulted. | Methods, 3.1 Search strategy: PubMed, Embase, Cochrane Library, Web of Science are listed. Search deadline: August 2025. |
| Search strategy | 7 | Present the full search strategies for all databases, registers and websites, including any filters and limits used. | Methods, 3.1 Search strategy: The search terms are mentioned and the complete strategy is described in Supplementary File 1 |
| Selection process | 8 | Specify the methods used to decide whether a study met the inclusion criteria of the review, including how many reviewers screened each record and each report retrieved, whether they worked independently, and if applicable, details of automation tools used in the process. | Methods, 3.3 Data extraction and quality assessment: It is mentioned that two reviewers screened independently, and differences were resolved by a third reviewer. |
| Data collection process | 9 | Specify the methods used to collect data from reports, including how many reviewers collected data from each report, whether they worked independently, any processes for obtaining or confirming data from study investigators, and if applicable, details of automation tools used in the process. | Methods, 3.3 Data extraction and quality assessment: It is mentioned that two reviewers extracted data independently, and differences were resolved by a third reviewer. |
| Data items | 10a | List and define all outcomes for which data were sought. Specify whether all results that were compatible with each outcome domain in each study were sought (e.g. for all measures, time points, analyses), and if not, the methods used to decide which results to collect. | Methods, 3.2 Selection criteria defined the primary outcome (permanent hypothyroidism). Methods, 3.3 Data extraction and quality assessment lists the extracted data items, including outcome incidence rates and effect estimates of predictors. |
|  | 10b | List and define all other variables for which data were sought (e.g. participant and intervention characteristics, funding sources). Describe any assumptions made about any missing or unclear information. | Methods, 3.3 Data extraction and quality assessment lists the extracted basic research information, participant characteristics and other variables. |
| Study risk of bias assessment | 11 | Specify the methods used to assess risk of bias in the included studies, including details of the tool(s) used, how many reviewers assessed each study and whether they worked independently, and if applicable, details of automation tools used in the process. | Methods, 3.3 Data extraction and quality assessment: Using the NOS scale, two reviewers assessed independently, and disagreements were resolved through discussion or by a third reviewer. |
| Effect measures | 12 | Specify for each outcome the effect measure(s) (e.g. risk ratio, mean difference) used in the synthesis or presentation of results. | Methods, 3.4 Statistical analysis: Pooled odds ratio (OR). Results, 4.3: Mean difference (MD) was used for FT3. |
| Synthesis methods | 13a | Describe the processes used to decide which studies were eligible for each synthesis (e.g. tabulating the study intervention characteristics and comparing against the planned groups for each synthesis (item #5)). | Results, 4.3: Describes which studies were included in each analysis (e.g. “A total of two studies were included in the analysis of TgAb status”). The selection criteria in the methods section provide the basis for grouping. |
|  | 13b | Describe any methods required to prepare the data for presentation or synthesis, such as handling of missing summary statistics, or data conversions. | Results, 4.3: It is mentioned that the units of FT3 data have been manually harmonized (“units of measurement had been manually harmonized”). |
|  | 13c | Describe any methods used to tabulate or visually display results of individual studies and syntheses. | Methods, 3.4 Statistical analysis mentions the use of RevMan and STATA. Figure legends mentioned forest plots and funnel plots. Study characteristics are summarized in Table 1. |
|  | 13d | Describe any methods used to synthesize results and provide a rationale for the choice(s). If meta-analysis was performed, describe the model(s), method(s) to identify the presence and extent of statistical heterogeneity, and software package(s) used. | Methods, 3.4 Statistical analysis: Describes the preferential use of random effects models, the use of I², Q test, τ² to assess heterogeneity, and the use of RevMan 5.4 and STATA 16.0 software. |
|  | 13e | Describe any methods used to explore possible causes of heterogeneity among study results (e.g. subgroup analysis, meta-regression). | Expectations of clinical heterogeneity are mentioned in the text (Results, 4.3). |
|  | 13f | Describe any sensitivity analyses conducted to assess robustness of the synthesized results. | Methods, 3.4 Statistical analysis and Results, 4.4: Describes sensitivity analysis using the “leave one out” method. |
| Reporting bias assessment | 14 | Describe any methods used to assess risk of bias due to missing results in a synthesis (arising from reporting biases). | Methods, 3.4 Statistical analysis and Results, 4.4: Describes the use of funnel plots and Egger’s test to assess publication bias. |
| Certainty assessment | 15 | Describe any methods used to assess certainty (or confidence) in the body of evidence for an outcome. | There is a textual evaluation in Discussion (“the certainty of evidence for most predictors is moderate to low”). |
| **RESULTS** | | |  |
| Study selection | 16a | Describe the results of the search and selection process, from the number of records identified in the search to the number of studies included in the review, ideally using a flow diagram. | Results, 4.1 and Figure 1 (PRISMA flow diagram) |
|  | 16b | Cite studies that might appear to meet the inclusion criteria, but which were excluded, and explain why they were excluded. | Results, 4.1 describes the number of exclusions and their reasons (e.g. inclusion criteria not met, data could not be extracted). |
| Study characteristics | 17 | Cite each included study and present its characteristics. | Results, 4.1 cited all 10 included studies. Table 1 presents the characteristics of each study in detail. |
| Risk of bias in studies | 18 | Present assessments of risk of bias for each included study. | Results, 4.2 and Table 2 (NOS score) |
| Results of individual studies | 19 | For all outcomes, present, for each study: (a) summary statistics for each group (where appropriate) and (b) an effect estimate and its precision (e.g. confidence/credible interval), ideally using structured tables or plots. | The effect estimates and confidence intervals for each study are shown in the forest plot in Figure 2 . Table 1 provides the sample size for each group (with/without permanent hypothyroidism). |
| Results of syntheses | 20a | For each synthesis, briefly summarise the characteristics and risk of bias among contributing studies. | Results, 4.3 The number and heterogeneity of included studies are described at the beginning of each analysis. An overall description of study characteristics and risk of bias is provided in 4.1 and 4.2. |
|  | 20b | Present results of all statistical syntheses conducted. If meta-analysis was done, present for each the summary estimate and its precision (e.g. confidence/credible interval) and measures of statistical heterogeneity. If comparing groups, describe the direction of the effect. | Results, 4.3: Pooled results for TgAb (OR, 95% CI, I², τ²), FT3 (MD, 95% CI, I²), and treatment modality (OR, 95% CI, I²) are reported. |
|  | 20c | Present results of all investigations of possible causes of heterogeneity among study results. | No relevant analyzes were performed and therefore no results can be reported. |
|  | 20d | Present results of all sensitivity analyses conducted to assess the robustness of the synthesized results. | Results, 4.4: Reports the results of the sensitivity analysis (robustness changes in association). Figures 3A, 3B show the results of the “leave one out” sensitivity analysis. |
| Reporting biases | 21 | Present assessments of risk of bias due to missing results (arising from reporting biases) for each synthesis assessed. | Results, 4.4: The p-value of Egger’s test is reported, indicating that no significant publication bias was found. Figure 3C, 3D shows the funnel plot. |
| Certainty of evidence | 22 | Present assessments of certainty (or confidence) in the body of evidence for each outcome assessed. | No structured tools were used for assessment. The Discussion section contains a narrative summary of the certainty of the evidence. |
| **DISCUSSION** | | |  |
| Discussion | 23a | Provide a general interpretation of the results in the context of other evidence. | Discussion, paragraphs 1, 2, and 3: Interpret the results of TgAb, FT3, and treatment methods in relation to existing evidence and pathological mechanisms. |
|  | 23b | Discuss any limitations of the evidence included in the review. | Discussion, fourth paragraph (“Several limitations must also be acknowledged...”): Factors such as the small number of studies, small sample sizes, and residual confounding are discussed. |
|  | 23c | Discuss any limitations of the review processes used. | Discussion, fourth paragraph: mentioned process limitations such as failure to perform stratified analysis by follow-up time and some predictive factors being too few to conduct meta-analysis due to too few studies. |
|  | 23d | Discuss implications of the results for practice, policy, and future research. | Discussion, the end of the first paragraph and the last paragraph: Implications for clinical risk stratification and follow-up strategies are mentioned, and the need for future research is implicit. |
| **OTHER INFORMATION** | | |  |
| Registration and protocol | 24a | Provide registration information for the review, including register name and registration number, or state that the review was not registered. | The end of Abstract and the last paragraph of Introduction: PROSPERO CRD420251064643. |
|  | 24b | Indicate where the review protocol can be accessed, or state that a protocol was not prepared. | The last paragraph of Introduction: Mention that the research protocol has been prospectively registered with PROSPERO |
|  | 24c | Describe and explain any amendments to information provided at registration or in the protocol. | “no major deviations from the registered protocol occurred” |
| Support | 25 | Describe sources of financial or non-financial support for the review, and the role of the funders or sponsors in the review. | Funding part: Supported by the National Natural Science Foundation of China. |
| Competing interests | 26 | Declare any competing interests of review authors. | Conflict of Interest section: Declaration of no conflict of interest. |
| Availability of data, code and other materials | 27 | Report which of the following are publicly available and where they can be found: template data collection forms; data extracted from included studies; data used for all analyses; analytic code; any other materials used in the review. | Data Availability section: Indicates that the data is included in the article and can be obtained from the corresponding author. |

*From:*  Page MJ, McKenzie JE, Bossuyt PM, Boutron I, Hoffmann TC, Mulrow CD, et al. The PRISMA 2020 statement: an updated guideline for reporting systematic reviews. BMJ 2021;372:n71. doi: 10.1136/bmj.n71. This work is licensed under CC BY 4.0. To view a copy of this license, visit <https://creativecommons.org/licenses/by/4.0/>
